# Amyloid and tau in the brain in sporadic Alzheimer’s disease: defining the chicken and the egg

**DOI:** 10.1007/s00401-014-1243-9

**Published:** 2014-01-23

**Authors:** Cheryl A. Hawkes, Roxana O. Carare, Roy O. Weller

**Affiliations:** Faculty of Medicine, Institute for Life Sciences, University of Southampton, Southampton, UK

In the October 2013 issue of *Acta Neuropathologica* there were three very interesting articles on: *Amyloid or tau: the chicken or the egg?* In the first article, David Mann and John Hardy [10] argued that the deposition of aggregated amyloid β (Aβ) protein in the brain is a primary driving force behind the pathogenesis of Alzheimer’s disease with tau pathology following as a consequential or at least a secondary event. In the communication that followed, Braak and Del Tredici [3] presented the contrary argument with accumulation of tau protein as the primary event in sporadic Alzheimer’s disease. Attems and Jellinger [2] questioned the concept of a chicken and egg and suggested that the majority of cases of age-associated dementia are not caused by one single primary pathological mechanism.

Many of the arguments put forward in these three contributions rely on observations derived from human brain material. Although human brain specimens have been essential for defining the diagnosis of Alzheimer’s disease and other dementias and for identifying some of the problems associated with those dementias, the study of human material is not necessarily the best and sole way of solving the “chicken and egg” problem. It is tacitly assumed that the primary problems lie with the deposition of insoluble Aβ as plaques in the brain, or the toxicity of soluble Aβ, or with the accumulation of hyperphosphorylated tau in neurons and neurites. But, *have we identified the egg? What is the primary problem?* In order to answer this question we should perhaps review the changes that occur in the brain with age and how they affect the pathophysiology of the brain and result in dementia.

One example of this approach would be to consider the major risk factors for sporadic Alzheimer’s disease viz: age and possession of the ε4 allele of apolipoprotein E (*APOE4*), and take the lead from observations in human brains to ask the questions “Why does Aβ accumulate in the brain with age?” “What are the pathophysiological consequences for the brain of the accumulation of Aβ in the walls of cerebral arteries and in brain parenchyma?” It is clear from the study of human brains that there is an age-related failure of elimination of Aβ. Experimental studies and observations in human brains suggest that various pathways for the elimination of Aβ from the brain fail with age. Those pathways include degradation by neprilysin [11] and other enzymes and absorption of Aβ into the blood [13, 19]. In addition, there is an age-related failure of elimination of Aβ along the perivascular drainage pathways [7] that serve as lymphatic drainage pathways for interstitial fluid and solutes (including Aβ) from the brain [4, 5, 17]. Impaired elimination of Aβ along perivascular drainage pathways is further accentuated in the presence of *APOE4* [8]. Hallmarks of such failure are the deposition of Aβ aggregates in the walls of arteries as cerebral amyloid angiopathy (CAA) and as plaques of Aβ in the brain parenchyma; there is also a rise in the level of soluble Aβ in the brain in Alzheimer’s disease [9, 15] and the accumulation of fluid in subcortical white matter as leukoaraiosis [12]. The significance of CAA has been emphasised in human and experimental studies which showed that severe CAA was strongly related to the presence of dementia [6, 18].

Two major causes of age-related failure of perivascular elimination of Aβ from the brain have been identified, first: the progressive stiffening that occurs in the walls of cerebral arteries with age [16]; second: the changes that occur in basement membranes with age [7]. Theoretical models suggest that the contrary wave that follows the pulse wave along cerebral arteries is a major motive force for perivascular drainage of Aβ from the brain [14]. According to this model, stiffening of cerebral arteries would reduce the amplitude of pulsations and thus reduce the motive force for drainage of Aβ. This is supported by experimental studies showing that a reduction in arterial pulsations impedes perivascular drainage [1]. Age-related changes in vascular basement membranes that are the pathways for perivascular drainage and in their component proteins have been detected in human cerebral arteries (see [7]); in mice such changes are associated with demonstrably impaired perivascular drainage [7]. Impairment and slowing of perivascular drainage is associated with the formation of fibrillar amyloid in the walls of cerebral vessels as CAA which further impedes perivascular drainage of Aβ [7]. Stiffening of cerebral artery walls and age-related changes in basement membranes appear to be universal factors in the failure of perivascular elimination of Aβ from the ageing brain and in Alzheimer’s disease.

What, therefore, is the *egg*? Age-changes in the walls of cerebral arteries that impair the drainage of soluble Aβ could well be a prime candidate [7]. The *chicken* that develops from this egg may be the accumulation of insoluble aggregates of Aβ as CAA and as plaques in the brain but it could also be loss of homoeostasis of the neuronal environment due to failure of elimination of a range of soluble metabolites from the brain parenchyma.

We have emphasised here that, despite the importance of studying the distribution of Aβ and tau in human post-mortem brain in dementias such as Alzheimer’s disease, it is equally important to consider the wider pathophysiological effects of ageing of cerebral arteries on the brain in relation to Alzheimer’s disease. Such changes may lead not only to the accumulation of Aβ in the brain and artery walls but also to loss of homoeostasis of the neuronal environment and disturbance of neuronal function that may be related to dementia. Facilitation of elimination of soluble Aβ and other metabolites from the brain along the walls of ageing arteries could be a fruitful therapeutic strategy for the prevention and management of Alzheimer’s disease [5].
